# Single-cell Transcriptome Analysis Identifies Senescent Osteocytes as Contributors to Bone Destruction in Breast Cancer Metastasis

**DOI:** 10.21203/rs.3.rs-4047486/v1

**Published:** 2024-03-14

**Authors:** Manish Adhikari, Japneet Kaur, Hayley M. Sabol, Aric Anloague, Sharmin Khan, Noriyoshi Kurihara, Marta Diaz-delCastillo, Christina Møller Andreasen, C. Lowry Barnes, Jeffrey B. Stambough, Michela Palmieri, Olivia Reyes-Castro, Elena Ambrogini, Maria Almeida, Charles A. O’Brien, Intawat Nookaw, Jesus Delgado-Calle

**Affiliations:** 1 Physiology and Cell Biology, University of Arkansas for Medical Sciences, Little Rock, AR, US.; 2 Division of Hematology and Oncology, Department of Medicine, Indiana University, Indianapolis, IN, US.; 3 Forensic Medicine, University of Aarhus, Aarhus, Denmark.; 4 Molecular Bone Histology lab, Department of Clinical Research, University of Southern Denmark, Odense, Denmark; 5 Department of Clinical Pathologyogy, Odense University Hospital, Odense University Hospital, Odense, Denmark.; 6 Department of Orthopedic Surgery; University of Arkansas for Medical Sciences, Little Rock, AR, US.; 7 Division of Endocrinology and Metabolism, University of Arkansas for Medical Sciences and Central Arkansas Veterans Healthcare System, Little Rock, AR, US.; 8 Department of Biomedical Informatics, University of Arkansas for Medical Sciences, Little Rock, AR, US.; 9 Winthrop P. Rockefeller Cancer Institute, University of Arkansas for Medical Sciences, Little Rock, AR, US.

**Keywords:** breast cancer, senescence, osteocytes, resorption, senolytics

## Abstract

Breast cancer bone metastases increase fracture risk and are a major cause of morbidity and mortality among women. Upon colonization by tumor cells, the bone microenvironment undergoes profound reprogramming to support cancer progression that disrupts the balance between osteoclasts and osteoblasts, leading to bone lesions. Whether such reprogramming affects matrix-embedded osteocytes remains poorly understood. Here, we demonstrate that osteocytes in breast cancer bone metastasis develop premature senescence and a distinctive senescence-associated secretory phenotype (SASP) that favors bone destruction. Single-cell RNA sequencing identified osteocytes from mice with breast cancer bone metastasis enriched in senescence and SASP markers and pro-osteoclastogenic genes. Using multiplex *in situ* hybridization and AI-assisted analysis, we detected osteocytes with senescence-associated distension of satellites, telomere dysfunction, and *p16*^*Ink4a*^ expression in mice and patients with breast cancer bone metastasis. *In vitro* and *ex vivo* organ cultures showed that breast cancer cells promote osteocyte senescence and enhance their osteoclastogenic potential. Clearance of senescent cells with senolytics suppressed bone resorption and preserved bone mass in mice with breast cancer bone metastasis. These results demonstrate that osteocytes undergo pathological reprogramming by breast cancer cells and identify osteocyte senescence as an initiating event triggering bone destruction in breast cancer metastases.

## Introduction

Bone metastases represent an advanced stage of breast cancer, marked by malignant cells escaping the primary breast tumor and colonizing bone tissue (1, 2). Skeletal metastases impact the overall course of the disease and are a major cause of morbidity, severe pain, impaired motility, pathologic fractures, and mortality (3). Existing work on skeletal metastasis has focused on how cancer cells benefit from the bone microenvironment by establishing a vicious cycle in which breast cancer cells reprogram osteoblasts or osteoclasts to cause aberrant bone destruction or bone formation, which in turn provide tumor cells with bone-derived growth factors that fuel tumor growth in bone (4, 5). Advances in the last decade provided a deeper understanding of the complexity of the tumor microenvironment in bone and led to the identification of new cellular interactions between cancer and bone cells contributing to cancer progression beyond those originally described in the “vicious cycle” paradigm (2, 6). Thus, understanding the interplay between cancer cells and other tumor microenvironment cells is critical to identifying new targets to interfere with bone metastasis.

Osteocytes, the primary resident cells of the bone tissue, have traditionally been recognized for their role in maintaining bone health and homeostasis (7) but have been overlooked in bone cancer research for decades. Recent evidence supports the idea that osteocytes are key players in the complex interplay between cancer and the skeletal system. Preclinical and clinical studies in multiple myeloma and metastatic prostate cancer demonstrate that osteocytes influence tumor growth, bone destruction, and even the efficacy of cancer therapies (8–13). Further, the knowledge acquired on the role of osteocytes and their derived factors in these cancers has provided the rationale for developing novel therapeutic interventions to treat cancer in bone (9, 14–16). In breast cancer metastases, mounting evidence suggests that osteocytes interact with cancer cells and can influence their proliferation, migration, and invasion abilities (17–20). However, how metastatic cancer cells impact osteocytes in the tumor microenvironment is largely unknown.

In this work, we utilized scRNA-seq and a combination of preclinical and clinical models to explore the impact of breast cancer bone metastasis on osteocytes *in vivo*. Our study shows that metastatic breast cancer cells induce premature senescence in osteocytes. Moreover, our results reveal that senescent osteocytes acquire a pro-osteoclastogenic senescence-associated secretory phenotype (SASP) that supports osteoclastogenesis and bone destruction. Further, we provide evidence that depleting senescent cells using senolytics may represent an attractive adjuvant therapy to blunt the bone loss in bones colonized by metastatic breast cancer cells.

## Results

### Breast cancer bone metastases induce a senescence gene expression signature in osteocytes.

**Figure 1.**
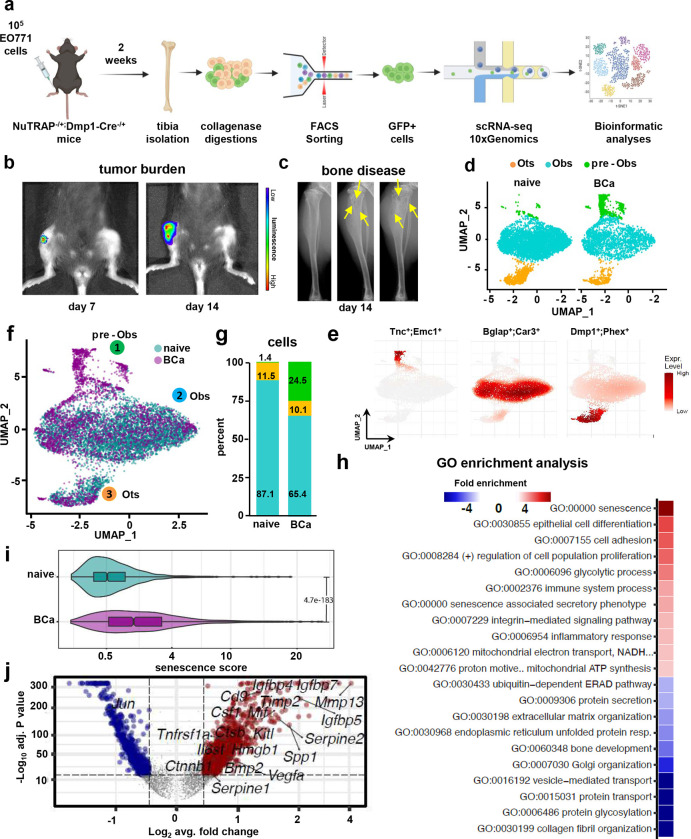
Single-cell transcriptomic profiling of osteoblastic cells in breast cancer bone metastasis. **(a)** Schematic of experimental design. **(b)** Tumor bioluminescence and **(c)** osteolytic lesions in X-Ray (yellow arrows) seven and fourteen days after tumor inoculation. Representative images per group are shown. Uniform Manifold Approximation and Projection (UMAP) plot representations of osteoblastic cells isolated from control (naïve) or mice with breast cancer bone metastasis (BCa) showing **(d)** three clusters: osteocytes (Ots), osteoblasts (Obs), and pre-osteoblasts (pre-Obs) and **(f)** cluster cell distribution by group. Each dot represents a single cell, and cells sharing the same color code indicate discrete populations of transcriptionally similar cells **(d)** or from the same group **(f)**. **(e)** Expression density plots with gene markers defining cluster identities. **(g)** The proportion of cells from each cluster in the naïve vs. BCa group. **(h)** Gene ontology (GO) enrichment analysis in genes differentially expressed between naïve vs. BCa osteoblastic cells. Positive values represent GO term enrichment in the BCa vs. naïve group. **(i)** Comparison of the senescence score in osteoblastic cells from naïve vs. BCa mice. **(j)** Volcano plot ranking genes according to their relative abundance (log2 fold change) and statistical value (−log10 p-value). Dots show significant upregulated (red) and down-regulated (blue) genes from naïve vs. BCa mice in osteoblastic cells.

To determine the impact of bone metastatic breast cancer cells on osteocytes, we crossed NuTRAP reporter mice (21), a Cre-inducible strain that allows labeling and simultaneous isolation of cell type-specific nuclei (mCherry) and mRNA (GFP), with Dmp1-8kb-Cre mice to induce active Cre recombination in osteocytes (22). We injected these mice intratibially with EO771-luc breast cancer cells (Fig. 1a). GFP^+^ cells were isolated two weeks after injection, when the mice displayed active tumor growth and established bone disease, and single-cell RNA sequencing (scRNAseq) was performed in the isolated cell population (Fig. 1b–c). Transcriptomic profiling of the GFP^+^ cells identified three distinct clusters: (1) pre-osteoblasts (pre-OBs), (2) osteoblasts (OBs), and (3) osteocytes (Ots) (Fig. 1d and **Suppl. Fig 1)**, defined by distinct gene expression patterns (Fig. 1e and **Suppl. Fig. 2)**. The osteocyte fraction represented ~10% of the total cells and remained unchanged between groups (Fig. 1f–g). In contrast, the osteoblast fraction decreased by 20%, and the pre-osteoblast fraction increased by 23% in the breast cancer vs. the control group (Fig. 1f–g). Apart from these three identified clusters, our analysis detected an additional cell type (FiX) characterized by high expression of *Acta2, Myh11, Tagln, Rgs5, and Igfbp7,* genes expressed by cancer-associated fibroblasts **(Suppl. Fig 3)** (23, 24). Intriguingly, this population expanded in bones with breast cancer bone metastasis **(Suppl. Fig 3)**. Next, we employed gene ontology (GO) analysis with the genes differentially expressed in cells isolated from control vs. breast cancer-bearing bones to identify signaling pathways altered in osteoblastic cells by breast cancer bone metastasis. When combining the three cell populations, we found enrichment in GO terms associated with cellular senescence, senescence-associated secretory phenotype (SASP), and inflammatory response (Fig. 1h). Further, cells isolated from bones with breast cancer metastasis exhibited a higher senescence score (constructed with previously reported transcriptional biomarkers of senescence (25, 26)), than from control bones (Fig. 1i) and upregulation of the SASP-related genes *Mmp13, Spp1, Serpine2, Timp2, Igfbp7, Igfbp5,* and *Vegfa*. (Fig. 1j).

**Figure 2.**
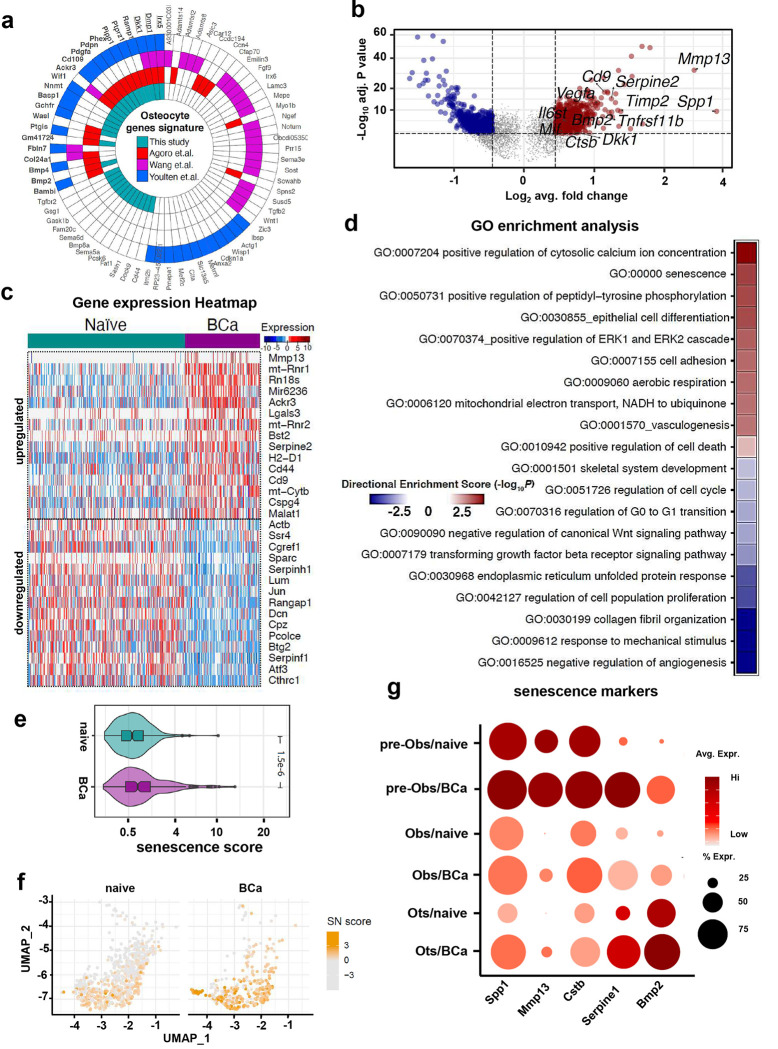
Osteocytes from bones with breast cancer tumors exhibit upregulation of genes associated with cellular senescence. **(a)** Polar plot showing osteocyte gene markers identified in our dataset compared to those previously reported by Agoro et al., Wang et al., and Youlten et al. Functional enrichment analysis results of Ots genes signature from different published resources. **(b)** Volcano plot ranking genes according to their relative abundance (log2 fold change) and statistical value (−log10 p-value). Dots show significant upregulated (red) and down-regulated (blue) genes in osteocytes from naïve vs. BCa mice. **(c)** Gene expression heatmap of the top 15 differentially expressed genes in osteocytes from naïve vs. BCa mice. **(d)** Gene ontology (GO) enrichment analysis in genes differentially expressed in osteocytes from control (naïve) vs. bones breast cancer tumors (BCa). Positive values represent GO term enrichment in osteocytes from the BCa vs. naïve group. **(e)** Comparison of the senescence score in osteocytes from naïve vs. BCa mice. **(f)** Uniform Manifold Approximation and Projection (UMAP) plot representations of osteocytes isolated from naïve or BCa mice showing the senescence score distribution by group. Each dot represents a single osteocyte, and the color intensity is proportional to the senescence score. **(g)** Bubble plot comparing expression of selected senescent markers in osteocytes (Ots), osteoblasts (Obs), and pre-osteoblasts (pre-Obs) from naïve vs. BCa mice. Bubble size is proportional to the percentage of cells in each cluster expressing a gene, and color intensity is proportional to average scaled gene expression within a cluster.

We next focused our analyses on the osteocyte population. Our dataset contains one of the largest osteocyte populations sequenced until now (719 vs. 340 osteocytes, naïve vs. breast cancer, respectively). The polar plot comparison displayed in Fig. 2a highlights common genes identified in our dataset and in previous studies, including the traditional markers *Dmp1, Phex, Dkk1,* and *Pdpn*, as well as new ones like *Cd44*, *Sema5a*, or *Tgfbr2*. We identified 850 genes differentially expressed in osteocytes from control vs. breast-cancer-bearing mice (Fig. 2b–c and Suppl. Table 1). In addition, GO term analysis uncovered upregulation of gene sets related to cellular senescence in osteocytes from bone metastases, whereas GO terms associated with cellular proliferation and cell cycle regulation were downregulated (Fig. 2d). Furthermore, we found an increased prevalence of osteocytes with a higher senescence score and expressing higher mRNA levels of the SASP-related genes *Spp1, Mmp13, Cstb, Serpine1,* and *Bmp2* (Fig. 2e–g). Similar observations were made in the OB and pre-OB clusters, which showed enrichment in senescence GO terms and senescence score and upregulation of SASP-related factors in bones from mice with breast cancer tumors **(Suppl. Fig. 4 and 5)**. Collectively, these results suggest that metastatic breast cancer generates a bone microenvironment conducive to premature cellular senescence in osteocytes and other osteoblastic lineage cells.

### Breast cancer cells promote cellular senescence and SASP in osteocytes.

**Figure 3.**
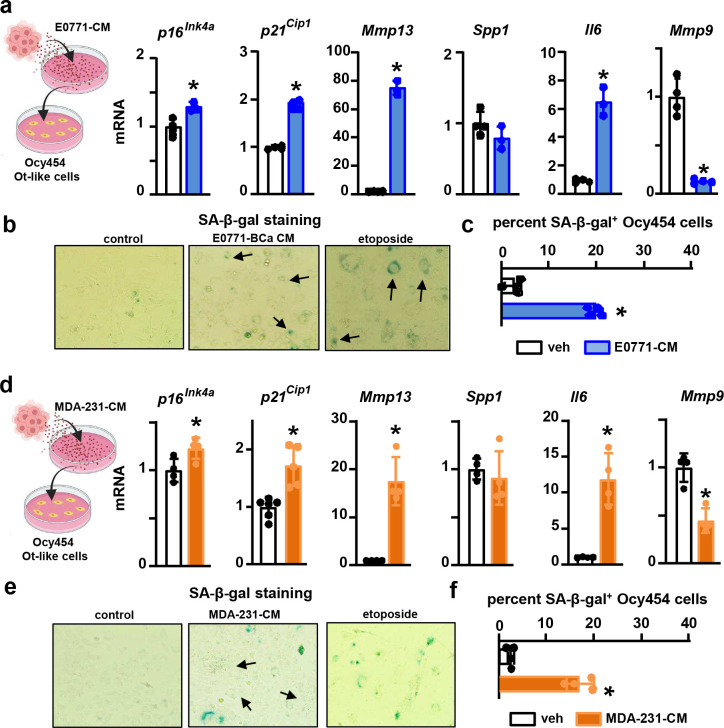
Breast cancer cells provoke cellular senescence in osteocyte-like cells. **(a)** Expression of senescence markers *p16*^*Ink4a*^ and *p21*^*Cip1*^ and SASP-related genes *Mmp13*, *Spp1*, *Il6*, and *Mmp9* and **(b)** representative images and (**c)** prevalence of SA-β-Gal^+^ cells in Ocy454 cells treated with vehicle or conditioned media (CM) from murine EO771 breast cancer cells for nine days. **(d)** Expression of senescence markers *p16*^*Ink4a*^ and *p21*^*Cip1*^ and SASP-related genes *Mmp13*, *Spp1*, *Il6*, and *Mmp9* and **(e)** representative images and (**f)** prevalence of SA-β-Gal^+^ cells in Ocy454 cells treated with vehicle or CM from human MDA-MB-231 breast cancer cells for nine days. n=3–4/group. *p<0.05 vs. vehicle by Student’s t-test. Data are shown as mean ± SD; each dot represents an independent sample; representative experiments out of two are shown.

To determine if breast cancer cells or their products can directly promote osteocyte senescence, we performed a series of *in vitro* studies using osteocyte-like cell lines and *ex vivo* cultures containing human and murine primary osteocytes. Conditioned media (CM) from murine EO771 or human MDA-MB-231 metastatic breast cancer cells markedly decreased cell number and modestly increased apoptosis in osteocyte-like cells **(Suppl. Fig. 6a-e)**. Inhibition of apoptosis using the *caspase3* inhibitor *DEVD* prevented apoptosis induced by EO771 cells but did not alter the number of live osteocytes. Together, these findings suggest that breast cancer cells provoke a proliferative arrest in osteocyte-like cells, a feature of senescence **(Suppl. Fig. 6f)**. Further, osteocyte-like MLO cell lines cultured in direct contact with breast cancer cells (24 hours) or treated with CM from breast cancer cells (48 hours) also had upregulation of senescence-related genes *p16*^*Ink4a*^*, p21*^*Cip1*^, *Mmp13,* and *Il6* **(Suppl. Fig. 7)**. Because MLO osteocyte-like cells are immortalized, they are not an optimal model for studying senescence. Thus, we used Ocy454 cells (27), conditionally immortalized when cultured at 33C, for the next *in vitro* studies. After two weeks at 37C, Ocy454 osteocyte-like cells exhibited morphological features and a gene expression profile consistent with mature osteocytes **(Suppl. Fig. 8)**. Ocy454 cells treated with CM from EO771 or MDA-231 breast cancer cells for nine days exhibited hallmarks of cellular senescence, including flattened and enlarged morphology (not shown), upregulation of the senescent markers *p16*^*Ink4a*^ and *p21*^*Cip1*^ and the SASP-related factor*s Il6* and *Mmp13* compared to control Ocy454 cells (Fig. 3a,d), high senescence-associated (SA)-β-Gal activity (Fig. 3b,e), and increased prevalence of SA-β-Gal^+^ cells (Fig. 3c,f). Remarkably, these features of senescence were already evident after 48 hours of treatment with CM from breast cancer cells **(Suppl. Fig. 9)**.

**Figure 4.**
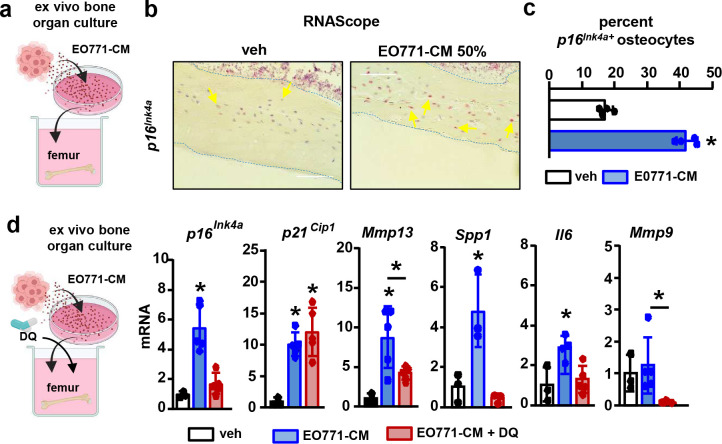
Metastatic breast cancer cells increase the expression of senescence markers and SASP factors in primary murine osteocytes. **(a)** Expression of senescence markers *p16*^*Ink4a*^ and *p21*^*Cip1*^ and SASP-related genes *Mmp13*, *Spp1*, *Il6*, and *Mmp9* and **(b)** representative images and **(c)** prevalence of *p16*^*Ink4a* +^ primary osteocytes in bones treated with vehicle or conditioned media (CM) from murine EO771 breast cancer cells cultured *ex vivo* for five days. **(d)** Expression of senescence markers and SASP-related genes in bones treated with vehicle or CM from murine EO771 breast cancer cells, in the presence/absence of the senolytics Dasatinib and Quercetin (DQ), cultured *ex viv*o for five days. n=3–5/group. *p<0.05 vs. vehicle by Student’s t-test **(a-c)** or vs. vehicle by One-Way ANOVA **(d)**. Data are shown as mean ± SD; each dot represents an independent sample; representative experiments out of two are shown.

**Figure 5.**
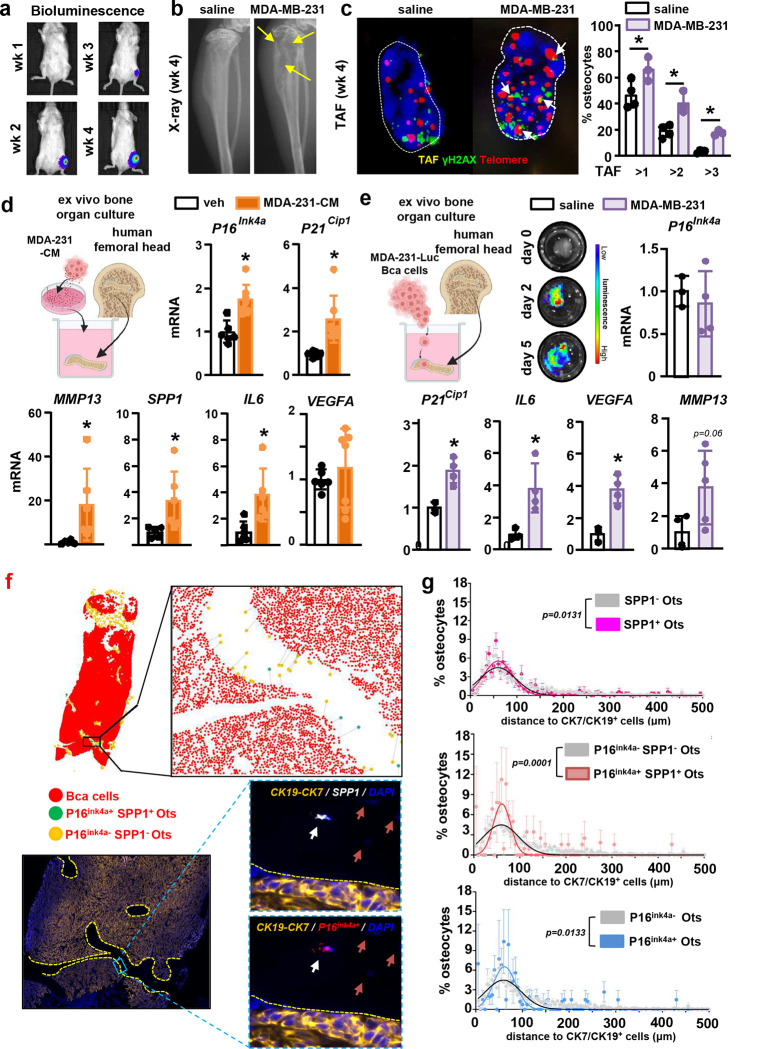
Infiltration of metastatic breast cancer cells upregulates senescence and SASP-related genes in human bones. **(a)** Representative *in vivo* bioluminescence images of mice with MDA-MB-231 breast cancer bone metastasis. **(b)** Osteolytic lesions in X-ray images (yellow arrows) 4 weeks after tumor inoculation. Representative images per group are shown. **(c)** Representative images and prevalence of telomere-associated foci (TAF)+ primary osteocytes in bones from naïve and MDA-MB-231 inoculated mice. White arrows indicate TAF events. White dashed lines indicate the nuclei’s contour. n=3–4 mice/group. **(d)** Expression of senescence markers and SASP-related genes in human bones treated with vehicle or CM from human MDA-MB-231 breast cancer cells cultured *ex vivo* for five days. **(e)** *Ex vivo* human bone-breast cancer cell organ cultures established with human MDA-MB-231-luciferase cancer cells and femoral head bone fragments from healthy human donors. Representative bioluminescence images of bones bearing MDA-MB-231 cells two and four days after cell implantation. Expression of senescence markers and SASP-related genes in human bones bearing human MDA-MB-231 breast cancer cells or saline cultured *ex vivo* for five days. n=3–5/group. *p<0.05 vs. vehicle by Student’s t-test. (**f**) Representative images of 1) a bone biopsy from a breast cancer patient with bone metastasis showing breast cancer cells (red), senescent osteocytes (green), and normal osteocytes (orange), 2) a blow-out of the AI-assisted analysis of the distance distribution from each osteocyte to the closest breast cancer cell in the marrow, and 3) a histological section stained for CK19-CK7 (orange), P16^*Ink4a*^ (red), SPP1 (white), and DAPI (blue). White arrows point to P16^*Ink4a*+^SPP1^+^ osteocytes, and red arrows point to P16^*Ink4*−^SPP1^−^ osteocytes. (**f**) AI-assisted quantitative analysis of the distance to breast cancer cells (CK7/CK19^+^) distribution for SPP1^+^, P16^*Ink4a*+^, P16^*Ink4a*+^SPP1^+^, and P16^*Ink4a*−^SPP1^−^ osteocytes, n=4. P values were calculated by the Kolmogorov-Smirnov test. Data are shown as mean ± SD; each dot represents an independent sample; representative experiments out of two are shown.

To study the responses of primary osteocytes to metastatic breast cancer cells, we next used *ex vivo* bone cultures (Fig. 4a), a system that recapitulates the spatial dimension, cellular diversity, and molecular networks of the tumor niche in a controlled setting (28). Treatment of bones with EO771-CM for two or five days increased the prevalence of *p16*^*Ink4a*+^ primary osteocytes by 30% vs. controls (Fig. 4b–c) and upregulated the expression of *p16*^*Ink4a*^*, p21*^*Cip1*^, *Mmp13, Spp1, Il6,* and *Mmp9* **(Suppl. Fig. 10a)**. Comparable results were observed with CM from human MDA-MB-231 breast cancer cells **(Suppl. Fig. 10b)**. Treatment with the senolytic drugs Dasatinib + Quercetin (DQ) prevented or reduced the increased expression of *p16*^*Ink4a*^*, Mmp13,* and *Il6* (Fig. 4d), supporting a direct link between the changes in gene expression and the cellular senescence induced by breast cancer cells. Next, we explored if human breast cancer metastasis increases the prevalence of senescent osteocytes *in vivo*. Consistent with our *in silico*, *in vitro*, and *in vivo* observations, bones bearing human MDA-MB-231 breast cancer bone metastasis exhibited a higher prevalence of telomere-associated-foci (TAF)^+^ senescent osteocytes (Fig. 5a–c). Moreover, we explored whether human breast cancer cells induce senescence in human osteocytes. MDA-231-CM upregulated *P16* ^*Ink4a*^*, P21*^*Cip1*^*, MMP13, SPP1,* and *IL6* gene expression compared to controls in human bones containing primary osteocytes (Fig. 5d). We also allowed breast cancer cells to infiltrate human bones cultured *ex vivo*. We detected active tumor growth after two days and a similar upregulation of senescence-related markers in bones infiltrated with metastatic breast cancer cells compared to control bones, except *P16*, which remained unchanged (Fig 5e). Lastly, we examined osteocyte senescence in bone biopsies from a small cohort of breast cancer patients with bone metastasis. We detected *P16*^*Ink4a*+^ and *SPP1*^+^ osteocytes (Fig. 5f), which preferentially located close to bone marrow areas infiltrated with breast cancer cells (Fig. 5g). The percent of *P16*^*Ink4a*+^*SPP1*^+^ osteocytes showed a non-significant positive correlation with tumor burden in the bone marrow (**Suppl. Fig. 11**). These findings, together with our bioinformatic results, demonstrate that metastatic breast cancer cells induce premature cellular senescence and SASP development in osteocytes.

### Senolytic therapy eliminates senescent osteocytes and mitigates bone loss in mice with breast cancer bone metastasis.

**Figure 6.**
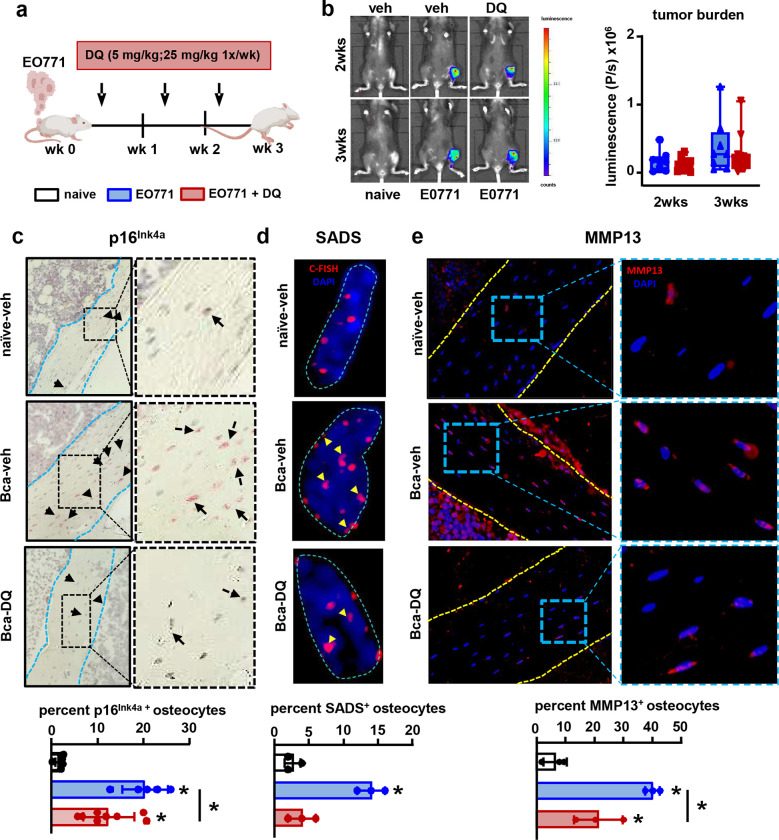
Senolytic therapy blunts the increase in senescent osteocytes in mice with breast cancer bone metastasis. **(a)** Experimental design. **(b)** Representative *in vivo* bioluminescence images and luminescence quantification in control mice (naïve) vs. mice with EO771 breast cancer bone metastasis (BCa) treated with vehicle (veh) or the senolytics Dasatinib and Quercetin (DQ). n=10 mice/group. **(c)** Representative images and prevalence of *p16*^*Ink4a*+^ primary osteocytes in bones from naïve and breast cancer mice receiving veh or DQ three weeks after tumor inoculation. Black arrows indicate *p16*^*Ink4a*+^ osteocytes. Blue dashed lines indicate the bone surface. n=5–8 mice/group. **(d)** Representative images and prevalence of senescence-associated distension of satellites (SADS)^+^ primary osteocytes in bones from naïve and BCa mice receiving veh or DQ three weeks after tumor inoculation. Yellow arrows indicate SADS events. Blue dashed lines indicate the nuclei’s contour. C-FISH: centromere-FISH. n=3 mice/group. **(e)** Representative images and prevalence of MMP13^+^ primary osteocytes in bones from naïve and BCa mice receiving veh or DQ three weeks after tumor inoculation. Yellow dashed lines indicate the bone surface. n=3/group. *p<0.05 vs. vehicle by One Way ANOVA. Data are shown as mean ± SD; each dot represents an independent sample.

**Figure 7.**
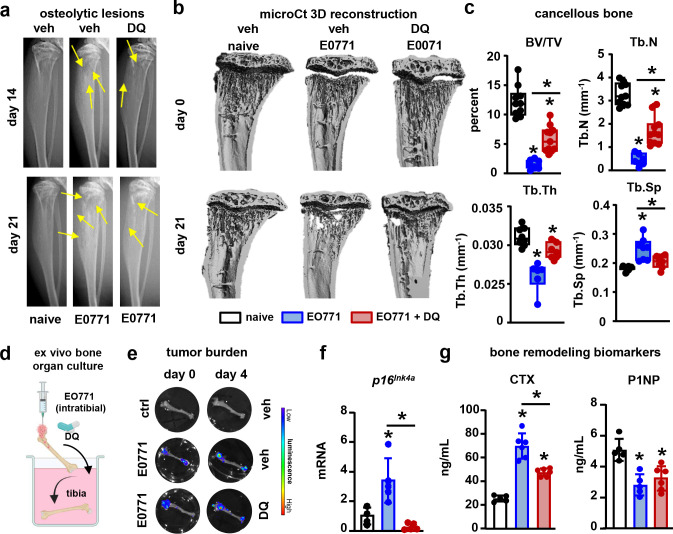
Pharmacologic depletion of senescent cells mitigates the osteolytic bone loss induced by breast cancer skeletal metastasis. **(a)** Representative X-ray longitudinal images of bones from control mice (naïve) and mice with EO771 breast cancer bone metastasis treated with vehicle (veh) or the senolytics Dasatinib and Quercetin (DQ). Yellow arrows indicate lytic lesions. **(b)** Representative microCT 3D reconstruction longitudinal images of tibiae and **(c)** cancellous bone mass and microarchitecture in bones from naïve and BCa mice receiving veh or DQ three weeks after tumor inoculation. Bone volume/tissue volume (BV/TV), trabecular number (Tb.N.), trabecular thickness (Tb. Th.), and trabecular separation (Tb. Sp.). n=10/group. **(d)** *Ex vivo* murine bone-breast cancer cell organ cultures established with murine EO771-luciferase cancer cells and murine tibias. **(e)** Representative bioluminescence images of tibiae bearing with EO771 cells four days after cell injection. **(f)** Expression of the senescence marker *p16*^*Ink4a*^ in bones injected with EO771 breast cancer cells or saline cultured *ex vivo* for five days in the presence/absence of DQ. **(g)** Level of the bone resorption marker (CTX) and the formation marker (P1NP) in the culture media of bones injected with EO771 breast cancer cells or saline cultured *ex vivo* for five days in the presence/absence of DQ. n=5–6/group. *p<0.05 vs. vehicle by One Way ANOVA. Data are shown as mean ± SD; each dot represents an independent sample.

Because the accumulation of senescent osteocytes has been linked to the bone loss seen with aging or radiation therapy (29, 30), we hypothesized that the accelerated cellular senescence induced by metastatic breast cancer cells in bone contributes to bone destruction. To test this hypothesis, we treated mice with DQ, a cocktail of the senolytic drugs known to deplete senescent bone cells (Fig. 6a) (29, 30), three days after injecting cancer cells intratibially. DQ therapy did not affect tumor progression (Fig. 6b). Bones-bearing breast cancer cells exhibited a higher prevalence of *p16*^*Ink4a*+^, senescence-associated distension of satellites (SADS)^+^, and MMP13^+^ osteocytes (Fig. 6c–e). Treatment with DQ prevented the increase in senescent SADS^+^ osteocytes and attenuated the increase in MMP13^+^ osteocytes in mice with bone metastasis (Fig. 6c–e). In addition, mice with breast cancer bone metastasis receiving DQ had fewer osteolytic lesions (Fig. 7a), higher bone mass, and improved bone microarchitecture than vehicle-treated mice with bone metastasis (Fig. 7b–c). At the end of the study, mice with bone tumors displayed a profound inhibition of bone formation and increased serum CTX levels **(Suppl. Fig. 12)**. At this time point, due to the aggressiveness of the model, we could not detect differences in CTX or bone formation rate between mice with bone metastasis receiving vehicle or DQ **(Suppl. Fig. 12)**, suggesting that the protective effects of DQ occurred during the initial stages of tumor progression. To assess this possibility, we developed an *ex vivo* model resembling the *in vivo* conditions in which tibiae from female mice were injected with EO771-luc cells and treated with vehicle or DQ (Fig. 7d). Like in the *in vivo* study, bones bearing breast cancer tumors exhibited *p16*^*Ink4a*^ mRNA upregulation, and higher CTX and lower P1NP in the culture media (Fig. 7e–g). Treatment with DQ did not affect tumor burden or P1NP levels; however, it restored *p16*^*Ink4a*^ expression to control levels and decreased by ~50% CTX levels in bones bearing breast cancer cells (Fig. 7f–g). These data suggest that breast cancer-induced senescence in the tumor niche is an initiating event that triggers bone loss in breast cancer bone metastasis by promoting bone resorption.

### Breast cancer-induced senescence increases the osteoclastogenic potential of osteocytes.

**Figure 8.**
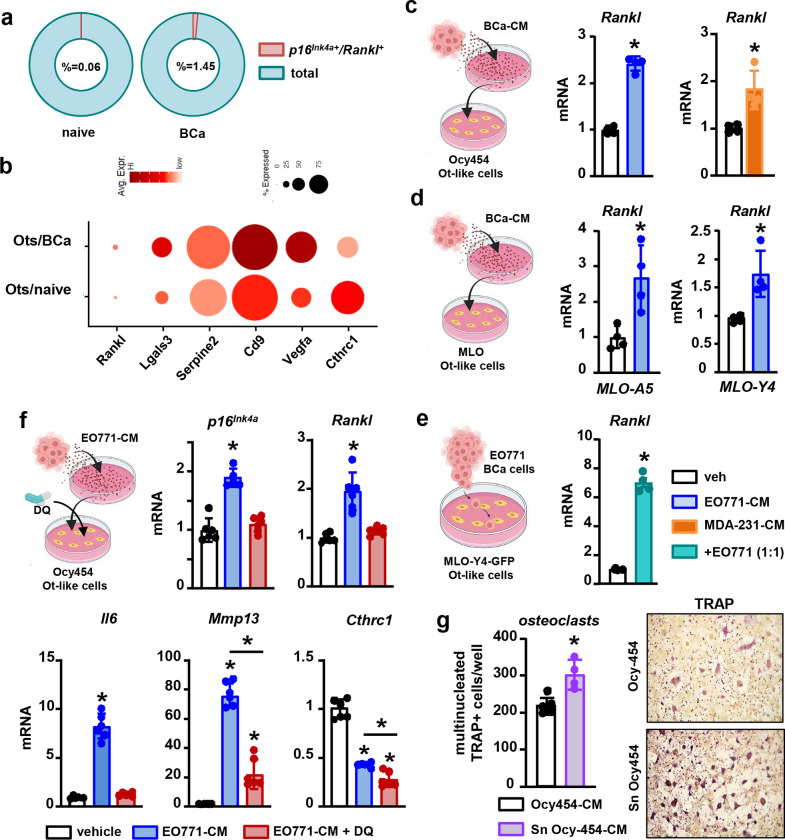
Breast cancer cells enhance osteocyte’s osteoclastogenic potential via cellular senescence. **(a)** Prevalence of double positive *p16*^*Ink4a*^-*Rankl* in cells isolated from control mice (naïve) and mice bearing breast cancer bone tumors (BCa) in the scRNAseq dataset. **(b)** Bubble plot comparing expression of selected pro-osteoclastogenic markers in primary osteocytes (Ots) isolated from naïve vs. BCa mice. Bubble size is proportional to the percentage of cells in each cluster expressing a gene, and color intensity is proportional to average scaled gene expression within a cluster. **(c-e)** *Rankl* expression in osteocyte-like cells (Ocy454, MLOA-5, and MLOY-4) treated with conditioned media (CM) from murine EO771 or human MDA-MB-231 breast cancer cells or cultured in direct contact with EO771 cells for two days. n=3/group. **(f)** Expression of *p16*^*Ink4a*^*, Rankl, Mmp13*, *and Cthrc1*in Ocy454 osteocytes treated with vehicle or CM from EO771 breast cancer cells cultured in the presence/absence of the senolytic agents Dasatinib and Quercetin (DQ) for two days. n=6/group. **(g)** Representative images and quantification of TRAP^+^ cells in pre-osteoclast cultures treated with CM from control or senescent Ocy454 osteocytes. n=4/group. *p<0.05 vs. vehicle by Student’s t-test **(c-e, and g)** or vs. vehicle by One-Way ANOVA **(f)**. Data are shown as mean ± SD; each dot represents an independent sample; representative experiments out of two are shown.

Further bioinformatic analyses of our scRNAseq data set revealed the presence of a higher number of *p16*^*Ink4a*+^*-Rankl*^+^ cells in bones with cancer metastasis, indicating the presence of senescent cells of the osteoblastic lineage with a pro-resorptive phenotype (Fig. 8a). Moreover, the comparative transcriptomic analysis identified that osteocytes from bones with breast cancer tumors had a gene signature associated with osteoclastogenesis, with changes in the expression of pro- and anti-osteoclastogenic factors *Rankl, Mmp13, Lgals3*, *Serpine3*, *Cd9*, *Vegfa*, and *Cthrc1* (Fig. 8b). Poised by these observations, we next investigated the effects of breast cancer cells on osteocyte-like cell lines. Treatment with breast cancer CM or direct co-culture with breast cancer cells upregulated *Rankl*, *Mmp13*, and *Il6* and decreased *Cthrc1* mRNA expression in MLO and Ocy454 cells (Fig. 8c–f). Treatment with DQ prevented *Rankl* and *Il6* upregulation and decreased by 80% the elevated *Mmp13* in Ocy454 cells treated with EO771-CM (Fig. 8f). Lastly, we investigated if premature cellular senescence in osteocytes contributes to osteoclastogenesis by assessing *in vitro* if factors derived from breast cancer-induced senescent osteocytes affect osteoclast precursor differentiation. We found that senescent osteocyte-CM led to the formation of more osteoclasts than CM from control Ocy454 cells (Fig. 8g). This set of experiments demonstrates that upon premature cellular senescence induced by metastatic breast cancer cells, osteocytes acquire a unique pro-osteoclastogenic SASP, which acts on osteoclast lineage cells in a paracrine manner to support osteoclastogenesis and bone destruction.

## Discussion

In this study, we mapped the transcriptome of osteocytes from normal bones and bones with breast cancer metastasis and defined a signature of 850 genes that distinguishes healthy from diseased osteocytes. We found that the transcriptome of osteocytes from bone colonized by breast cancer cells is significantly enriched in genes associated with cellular senescence. Further, we demonstrate that breast cancer cells induce premature cellular senescence and a distinctive pro-osteoclastogenic SASP in osteocytes using various mouse models and histological sections from breast cancer patients with bone metastasis. Moreover, we show that senescent osteocytes promote osteoclastogenesis and that pharmacological depletion of senescent cells mitigates bone resorption and improves bone mass and microarchitecture in a mouse model of bone metastatic breast cancer. Collectively, these findings unravel the profound cellular and molecular reprogramming that osteocytes undergo in breast cancer metastasis and illustrate the contribution of osteocytes to the lytic bone disease seen in breast cancer metastasis.

scRNAseq has emerged as a transformative tool in bone research, enabling a detailed exploration of the gene expression profiles at the individual cell level. However, previous efforts have failed to capture significant numbers of osteocytes. Our study is one of the first to leverage scRNAseq to study the osteocyte transcriptome *in vivo* in the dynamic context of breast cancer bone metastasis. Our approach using a NuTRAP reporter mouse, a novel tool in bone cell studies, crossed with the Dmp1-8kb-Cre strain, allowed us to achieve the isolation and sequencing of a significantly higher number of osteocytes compared to other methods (TdTomato or microbeads) (31–33). Through comparative analysis with existing datasets, this study begins to delineate a set of common markers defining primary osteocytes under physiological conditions, which goes beyond the more traditional markers previously described (34). In addition, our findings reveal a distinctive gene signature of diseased osteocytes specific to breast cancer bone metastasis, a critical step to understanding the molecular intricacies involved in the reprogramming of osteocytes by breast cancer cells. Our method also captured osteoblast and pre-osteoblast populations and unraveled an accumulation of osteoblast precursors accompanied by a reduction in osteoblasts in bones with breast cancer metastases. These *in vivo* results corroborate previous *in vitro* findings showing that breast cancer cells suppress the differentiation of osteoblast precursors (16, 35–38), which might accumulate in the bone marrow niche over time. The bioinformatic analysis of our dataset also unveiled a new cell population descended from Dmp1^+^ cells characterized by high expression of genes associated with cancer-associated fibroblasts and increased in bones colonized by breast cancer cells. Future studies beyond the scope of the current work are warranted to investigate the biological implications of this cell population.

The current study shows that metastatic breast cancer cells in bone promote a rapid increase in senescent osteocytes. Cellular senescence is a cell fate that involves irreversible proliferative arrest, altered chromatin organization, and resistance to apoptosis. We used a rigorous orthogonal approach to confirm the presence of osteocyte senescence. First, we generated a bioinformatic senescence score algorithm, allowing for a more comprehensive assessment of senescent cells at the transcriptome level. This approach bypasses the limitations of relying solely on the expression of the p16^Ink4a^ transcript levels to identify senescent cells due to its low and variable expression in senescent cells (25). Second, we confirmed the utility of our bioinformatic tool to predict senescence using *in vitro*, *in vivo*, and novel *ex vivo* models of breast cancer bone metastasis. Consistent with the elevated senescence score in osteocytes from breast cancer bone metastasis, we detected an accumulation of senescent osteocytes using a combination of gold standard methods to detect senescence: SA-β-Galactosidase staining, in situ hybridization (*p16*^*Ink4a*^), immunostaining (SASP), and FISH (SADS/TAF) approaches. Lastly, we used AI-assisted histological phenotyping to confirm the presence of senescent osteocytes close to areas of the bone marrow colonized with metastatic breast cancer cells in patients. This combination of *in silico*, preclinical, and clinical data shows that our bioinformatic senescence score is a powerful tool to assess cellular senescence in transcriptomic datasets and demonstrates that osteocyte cellular senescence is prematurely induced by breast cancer cells in the tumor niche.

Accumulation of senescent osteocytes has been described in various models of bone loss induced by aging, radiotherapy, or diabetes (29, 30, 39). In these models, senolytics or osteocyte-specific genetic depletion of senescent cells improve bone mass by suppressing bone resorption and maintaining/increasing bone formation (30, 40). Our work shows that senolytic therapy also protects from bone loss in breast cancer metastasis. Our *in vivo* and *in vitro* studies support that, in the context of breast cancer, senescent osteocytes have increased osteoclastogenic potential (increased *Rankl*, *Mmp13*, *Il6*) and are key drivers of cancer-induced bone resorption. This observation coincides with a prior study showing that senescent osteoblastic cells have increased osteoclastogenic potential and overexpress RANKL in the context of skeletal aging (41). While our research demonstrates that senescence plays a significant role in driving these transcriptional changes, additional factors, including PTHrP produced by breast cancer cells, are likely to play a contributory role in enhancing the osteoclastogenic potential of osteocytes, irrespective of cellular senescence (42). In contrast, senolytic therapy did not affect bone formation, suggesting that other mechanisms different from cellular senescence are responsible for the reduced osteoblast function caused by metastatic breast cancer. Notably, global genetic clearance of senescent cells results in greater benefits than osteocyte-specific clearance in aging, suggesting the involvement of additional senescent cell populations in bone preservation (40). Thus, we cannot exclude the possibility that in addition to osteocytes, clearance of other senescent cell populations (osteoblasts or pre-osteoblasts) identified in our study also contributes to the reduced bone loss seen with senolytics. Further studies are warranted to determine the specific contribution of senescent osteocytes versus other senescent cells in the tumor niche.

Many chemotherapeutic interventions trigger senescence in cancer cells, producing stable cell cycle arrest and reducing tumor growth. However, this process eventually leads to SASP induction and the creation of a pro-inflammatory and immunosuppressive microenvironment that can support tumor progression (43–46). Senolytics have anti-tumor efficacy in cancer cell lines when combined with various senescence-inducing chemotherapies (43–46). Further, high doses of senolytic agents alone can decrease breast cancer cell proliferation and tumor growth, although the results reported appear to be concentration- and cell-dependent (47–49). Moreover, genetic or pharmacologic induction of senescence in osteoblastic cells, stromal cells, or fibroblasts within the tumor microenvironment has been shown to promote breast cancer growth and bone disease (50, 51). This body of work suggests that inhibition of cellular senescence could affect tumor growth via direct or indirect mechanisms. Similar to a recent study using the senolytic ABT-263 (52), we found that treatment with DQ did not affect tumor growth in bone. The absence of an anti-tumor effect can be attributed to either the dose of senolytic therapy used in our studies or the inability of malignant tumors to undergo spontaneous senescence without external interventions such as chemotherapy or radiation therapy (53). Therefore, additional research is needed to determine if senescent osteocytes affect tumor progression in bone. Additionally, future studies are warranted to investigate whether senolytics maintain their ability to preserve bone while reducing tumor growth in a ‘one-two punch’ sequential treatment approach—employing senescence-induced chemotherapy followed by senolytic therapy.

In summary, by combining transcriptomic, bioinformatic, and pharmacologic approaches, we identified that metastatic breast cancer cells transcriptionally reprogram and induce premature senescence in osteocytes. The results of our study extend beyond existing work focusing on the interactions between metastatic breast cancer cells and osteoblasts/osteoclasts by shedding light on the pivotal role of senescent osteocytes as mediators of bone resorption in metastatic breast cancer. In addition, this work identifies cancer-induced senescence as an initiating event in bone metastases, and underscores the therapeutic potential of targeting senescence cells, including osteocytes, in the tumor niche to mitigate bone loss in cancer patients with bone metastasis.

## Materials and Methods

### Reagents.

Liberase was purchased from Roche (Mannheim, Germany). DPBS, HBSS, DAPI, and Trizol were purchased from Thermofisher Scientific (Waltham, MA, US). Dasatinib (cat #D-3307) was ordered from LC laboratories (Woburn, MA, US) and Quercetin (cat#Q4951) from Sigma Aldrich (St. Louis, MO, US). Annexin V-PI apoptosis kit was obtained from BD Biosciences (Franklin Lakes, NJ, US). Dulbecco’s Modified Eagle Medium (DMEM), α-Minimum Essential Media (α-MEM), fetal bovine serum, bovine calf serum, antibiotics (penicillin/streptomycin), and TriZol were purchased from Life Technologies (Grand Island, NY, US). Trypan Blue was purchased from Sigma Aldrich. Normocin, Plasmocin, and Puromycin (Cat#58-58-2) were obtained from InvivoGen (San Diego, CA, US). D-Luciferin was obtained from Perkin Elmer (Houston, TX, US) and Coelenterazine from Nanolight Technology (Pinetop, AZ, US).

### Cell culture.

Murine EO771 (RRID:CVCL_GR23) and EO771-luciferase (EO771-luc) expressing mammary cancer cells were provided by Dr. Karbassi (University of Arkansas for Medical Sciences, AR, US) and Dr. Norian (University of Alabama at Birmingham, AL, US), respectively. Human MDA-MB-231 (RRID:CVCL_0062) breast adenocarcinoma cells were purchased from ATCC (Manassas, VA, US). MDA-MB-231-luciferase cells for *ex vivo* experiments were generated by transducing the cells with lentiviral particles carrying a non-secreted Gaussia luciferase vector purchased from BPS Bioscience (Cat# 79893-C; San Diego, CA US). MDA-MB-231-luciferase cells for *in vivo* experiments were provided by Dr. Zhang (Baylor College of Medicine, TX, US). All breast cancer cells were cultured in DMEM with 10% FBS, 1% penicillin and streptomycin, 0.2% Normocin, 1.5mg/ml sodium bicarbonate, and 2% HEPES buffer. Murine Ocy454 (RRID:CVCL_UW31) osteocyte-like cells were provided by Dr. Pajevic (Boston University, MA, US) (27) and cultured in α-MEM medium with 10% FBS, 1% penicillin and streptomycin, and 0.2% Normocin on rat type I collagen-coated flasks. Prior to performing the experiments, Ocy454 cells were cultured at 37°C for two weeks. Murine MLOA-5 (RRID:CVCL_0P24) and MLOY-4 (RRID:CVCL_M098) osteocyte-like cells were obtained from Kerafast (Boston, MA, US) and cultured in 2.5% FBS and 2.5% BCS with 1% penicillin and streptomycin and 0.2% Normocin. MLO-Y4-GFP cells were described before (54). Cell lines were routinely assessed for mycoplasma and authenticated by morphology, gene expression profile, and tumorigenic capacity. Cell culture studies were performed by 1) treating breast cancer cells or osteocytes with conditioned media (CM) (50%) from breast cancer or osteocyte-like cells for 48 hours or 2) co-culturing breast cancer and osteocyte cells in a cell-to-cell manner (1:1) for 24 hours. Breast cancer and osteocytic CM were prepared by culturing 2×10^6^ cells in 10ml of culture medium for 48 hours. Osteocyte-like cultures were treated with Dasatinib (200 nM) and Quercetin (50 μM) after 48 hours of incubation with breast cancer CM.

### Animals studies.

We generated NuTRAP^−/+;^DMP1-8kb-Cre^−/+^ reporter mice by crossing B6;129S6-Gt(ROSA)26Sortm2(CAG-NuTRAP)Evdr/J mice (NuTRAP; #029899; Jackson Laboratory, ME, US) (21) with DMP1-8kb-Cre mice (55). 7-week-old NuTRAP^−/+;^DMP1-8kb-Cre^−/+^ female mice were inoculated intratiabilly with 10^5^ EO771-luc cells or PBS as control and sacrificed after 14 days. 7-week-old NOD.Cg-*Prkdc*^*scid*^ *Il2rg*^*tm1Wjl*^/SzJ mice were injected with 105 MDA-luc cells or saline and sacrificed after 4 weeks. 7-week-old C57BL/6 female mice were injected intratibially with 10^5^ EO771-luc cells or saline and three days later randomized by body weight to the following groups: 1) naïve mice orally receiving vehicle (10 % EtOH, 30 % PEG, 60 % Phosal-50 PG), 2) EO771-luc-bearing mice orally receiving vehicle, or 3) EO771-luc-bearing mice orally receiving a senolytic cocktail (DQ) of Dasatinib (5mg/kg) and Quercetin (50 mg/kg) once a week. The sample size was calculated based on previous studies (9, 14). Mice were housed in ventilated cages and maintained within a pathogen-free, accredited facility under a 12h light–dark cycle with constant temperature (23°C) and access to food and water ad libitum.

### 10X Genomics Single-cell RNA sequencing.

Cells were isolated from the tibias of NuTRAP^−/+^; DMP1-8kb-Cre^−/+^ mice after removing the muscle, periosteum, and epiphyses two weeks after saline/breast cancer cell injection. Next, we flushed out the bone marrow and performed serial Liberase/EDTA digestions as described before (56). Cells from the digestions were pooled, and GFP^+^ cells were FACS-sorted. Cells per condition were encapsulated using a Chromium Controller (10X Genomics, Pleasanton, CA, US), and libraries were constructed using a Chromium Single Cell 3’ Reagent Kit (10X Genomics) by the UAMS Genomics Core. The libraries were sequenced using an Illumina NovaSeq 600 machine to generate fastq files. Three independent samples were sequenced for the breast cancer group **(Suppl. Fig 1)** and pooled for the final bioinformatic analysis.

### Bioinformatic analyses.

The fastq files were preprocessed using Cell Ranger software version 6 (10X Genomics) to produce feature-barcode matrixes. The alignments were performed using mouse reference genome mm10 and imported for further analysis into the R suite software environment using Seurat package v4.2.0 (57–59). Quality control protocols were applied to remove outlier barcodes based on depth, number of genes, and proportion of mitochondrial genes. Harmonization/integration of different samples was performed using the reciprocal PCA method based on dimensional reduction using UMAP (58). Subpopulation identification and clustering were performed using the Louvain algorithm with multilevel refinement (60). The gene-specific markers of individual clusters were identified using the function FindMarkersAll using the MAST algorithm for cell type identification (61). Function/pathway enrichment analysis will be performed using PIANO (62). The Senescence and SASP GO terms were created by compiling the publicly available datasets of differentially regulated genes in senescent cells (25, 26). Senescence gene sets from refs. (25, 26) were used to calculate a gene signature score using PIANO (61). A Z-score was used to calculate statistical differences. The polar figure was generated using publicly available datasets from Agoro et al., Wang et al., and Youlten et al. (31, 32, 63).

### *Ex-vivo* bone culture.

*Ex-vivo* murine bone cultures were established with femurs from C57BL/6 female mice (EO771 cells) or NOD.Cg-Prkdcscid Il2rgtm1Wjl/SzJ (NSG; #005557, Jackson’s Lab) female mice (MDA-MB-231). We 1) injected 10^5^ EO771 breast cancer cells on femoral bones or 2) treated femurs with 50% CM from breast cancer cells in the presence/absence of Dasatinib (400 nM) and Quercetin (100 μM) for up to 5 days. Senolytics were added on day 0 and refreshed on day 3. *Ex vivo* human bone organ cultures were established with human cancellous bone fragments similar in size obtained from the femoral head discarded after hip arthroplasty. The bone samples were obtained from 2 females with no pathologies or medications that could affect bone mass or architecture. For these *ex vivo* cultures, we 1) plated 2×10^5^ MDA-MB231 breast cancer cells on human bones or 2) treated them with 50% MDA-MB-231 CM for five days. The CM was refreshed every 72 hours. Tumor bioluminescence was imaged 10 min after incubating bones with D-luciferin (150 μg/ml) or coelenterazine (100 μM) using an IVIS lumina XRMS system (Perkin Elmer, MA, US).

### Apoptosis and proliferation assays.

Cell proliferation/death/apoptosis were estimated using the Trypan Blue exclusion method as previously described, by flow cytometry using the Annexin V apoptosis Detection kit following the manufacturer’s recommendations, or by chromatin condensation and nuclear fragmentation of cells transfected with nuclear green fluorescent protein (8, 10). To block apoptosis, osteocyte-like cells were pre-treated with 50 nmol/L of the caspase inhibitor DEVD (Sigma-Aldrich, St. Louis, MO, US) 1 hour before the addition of breast cancer CM. DEVD was refreshed every 24 hours.

### Osteoclastogenesis.

Ocy454 osteocyte-like cells were cultured with 50% EO771-CM for nine days. Cells were washed two times with PBS, and fresh culture media was added. After 48 hours, CM was harvested from control and senescent Ocy454 cells. CD11b^+^ mononuclear cells were cultured in α-MEM containing 10% FBS plus 10 ng/mL of M-CSF for three days and then treated with a suboptimal dose of RANKL (10 ng/mL) for four days in the presence/absence of 25% CM from control and senescent osteocytes. RANKL and CM were replenished every two days. Cells were stained for TRAP using a leukocyte acid phosphatase kit (Sigma-Aldrich), and TRAP-positive mononuclear cells and multinuclear cells (≥3 nuclei/cell) were scored as described before (8).

### Senescence-associated beta-galactosidase (SA-β-Gal) staining.

Osteocyte-like cells were treated with 50% of CM from breast cancer cells for 2 to 9 days or Etoposide (10uM) for four days. After incubation, cells were washed with PBS, fixed, and stained with SA-β-Gal staining solution: (1 mg/ml X-Gal, cat# X1220, Teknova, California, US), 40 mM citric acid, pH 6.0, 5 mM potassium ferrocyanide, 5 mM potassium ferricyanide, 150 mM NaCl, 2 mM MgCl2) at 37 °C for 16–18 hrs (41). The number of positive/negative SA-β-Gal cells was imaged and quantified using a bright field microscope (EVOS FL Auto) at 20x. Three random areas from each well were imaged and quantified per group in a blinded fashion by two independent investigators.

### Gene expression.

Total RNA was isolated from cells and bone tissues using Trizol and converted to cDNA (Applied Biosciences), following the manufacturer’s directions*.* Gene expression was quantified by quantitative real-time PCR (qPCR) using TaqMan assays from Applied Biosystems, following the manufacturer’s directions. MLOY-4 GFP^+^ cells were co-cultured with EO771 breast cancer cells in a 1:1 ratio for 24 hours and sorted using a FACS sorter (FACSAria III, BD Biosciences, Franklin Lakes, NJ, US). Gene expression levels were calculated using the comparative threshold (CT) method and were normalized to the housekeeping gene GAPDH (8, 10). Fold changes were calculated using the control/vehicle conditions as the reference.

### RNA in-situ hybridization (RNAscope).

RNA in situ hybridization was performed using the RNAScope 2.5 HD detection reagent RED kit from Advanced Cell Diagnostics (Newark, CA, US) following the manufacturer’s instructions, as previously described (64). The following probes were incubated on paraffin-embedded tissue sections for 2 hours at 40C: murine *p16*^*Ink4a*^ (Cat#411011) and positive/negative controls (Cat#313911, Cat#310043). The signal was detected for 10 min at RT. Sections were counterstained with hematoxylin, dehydrated at 60C for 20 min, and mounted with VectaMount permanent mounting medium (Vector Laboratories, Newark, CA, US). The number of positive/negative osteocytes was quantified using a brightfield microscope at 40X magnification. Analyses were performed in the cortical bone of an 800-μm region of the tibia, starting 200 μm below the growth plate, in a blinded fashion by two independent investigators.

### Senescence-associated distension of satellites (SADS) analysis of senescent osteocytes.

The pericentromeric satellite heterochromatin undergoes decondensation and elongation in senescent cells, and the large-scale unraveling of pericentromeric satellite heterochromatin DNA, termed SADS, is a well-established marker of cell senescence *in vivo* (65). We used non-decalcified tibiae from naive and breast-cancer-bearing mice embedded in methylmethacrylate (MMA). Fluorescent *in situ* hybridization staining was performed as previously described (66). SADS were visualized in osteocytes (senescent osteocytes ≥4 SADS per osteocyte) using c-FISH (Cy3-labeled (F3002), CENPB-specific [ATTCGTTGGAAACGGGA] peptide nucleic acid (PNA) probe (Panagene Inc, Korea) and quantified by confocal microscopy (Zeiss LSM 880, 100x oil) in the cortical bone starting 200 μm below the growth plate. At least 50 nuclei were analyzed for each sample in a blinded fashion by two independent investigators.

### Telomere dysfunction-associated foci (TAF) assay.

TAF assays were performed on murine non-decalcified tibias embedded in methyl-methacrylate using a protocol established by Farr and Khosla labs (39). TAFs were visualized in osteocytes (senescent osteocytes ≥3 TAFs per osteocyte) using a primary antibody for γ-H2AX (1:200; anti–γ-H2A.X rabbit monoclonal antibody, Cell Signaling Technology; 9718) and Cy3-labeled telomere-specific (CCCTAA) peptide nucleic acid probe (TelC-Cy3, Panagene Inc.; F1002). The mean number of TAF per osteocyte was quantified by confocal microscopy (Zeiss LSM 880, 63x oil) in the cortical bone starting 200 μm below the growth plate. At least 35 nuclei were analyzed for each sample in a blinded fashion by two independent investigators.

### Immunofluorescence.

MMP13 immunofluorescence staining was performed on decalcified paraffin-embedded bone tissue sections. Tissue sections were incubated with anti-MMP13 (1:50; Abcam, AB39012) overnight at RT, washed, and incubated with Goat Anti-Rabbit IgG H&L – Alexa Fluor 594 (1:1000; Abcam, AB150080) for 1 hour and with copper sulfate (cat# 209198, Sigma-Aldrich) for 10 minutes, and mounted with Prolong Gold Anti-Fade 4’,6-diamidino-2-phenylindole (DAPI) mounting medium (cat# P36935, Invitrogen). MMP13 positive/negative osteocytes were imaged using Zeiss Axio Imager.M2 image system at 40X magnification. The percentage of MMP13-positive osteocytes was assessed using Fiji (67) in a blinded fashion by two independent investigators.

### Bone histomorphometry.

Static and dynamic bone histomorphometric analyses were performed using the OsteoMeasure High-Resolution Digital Video System (OsteoMetrics, Decatur, GA, US) as previously described (9, 14). Analyses were performed in the cancellous bone of an 800-μm region of the tibiae, starting 200 μm below the growth plate.

### Serum Biochemistry.

The bone resorption biomarker C-telopeptide of type 1 collagen (CTX) (Immunodiagnostic Systems, Cat#AC-06F1) and the bone formation marker propeptide of type 1 collagen (P1NP) (Immunodiagnosticsystems; Cat#AC-33F1) were analyzed in serum from mice or in conditioned media from bones cultured *ex vivo*, as previously described (9, 14).

#### Analysis of skeletal phenotype:

Osteolytic lesions were imaged using a Faxitron X-ray radiography system (Hologic, Marlborough, MA, US) as previously described (9, 14). MicroCT imaging was performed in live mice using a vivaCT 80 (Scanco Medical AG, Switzerland). Analyses were performed at the cancellous bone of the proximal tibia, in an area 20 μm below the growth plate, using 10 μm resolution.

### Bioluminescence.

Tumor growth was monitored weekly using an IVIS lumina XRMS system. Mice were injected with 150 mg/kg of D-luciferin intraperitoneally, and luminescence imaging was initiated 10 min after luciferin injection.

### Patient cohort and AI-assisted histological analysis.

Archived diagnostic transiliac biopsies collected at the Pathological Biobank at Odense University Hospital, Denmark, from four consented female patients with breast cancer metastatic bone disease were used for this analysis. The average patient age was 63 years (range 51–73 years old), and all patients received radiotherapy for their primary cancer and zoledronic acid at the time of dissemination to bone (4–5 mg/year, one dose). None of the patients received chemotherapy within the last 5 years before dissemination. 3-mm bone biopsies were fixed in 4% formalin for 24 h, decalcified in 10% formic acid for 7h, and embedded in paraffin. One 3.5-μm-thick section from each sample was multiplex immunostained for cytokeratin 7 and 19 (CK7 and CK19) along with fluorescent *in situ* hybridization for *SPP1* and *CDKN2A* expression. Briefly, sections were deparaffinized in a xylene and ethanol gradient and pre-treated in Custom Reagent (Advanced Cell Diagnostics, Hayward, CA, USA) for 20 min at 40°C. Sections were then hybridized overnight at 40°C with a channel 1 probe targeting the 6–1500 nucleotide region of *SPP1* mRNA (NM_001251829.1) and a channel 2 probe targeting nucleotides 95–1206 of *CDKN2A* mRNA (NM_000077.4). Signal amplification was performed per manufacturer recommendations and visualized with opal 690 and opal 620 dyes (Akoya Biosciences, Marlborough, MA, USA). Sections were then HRP blocked (Advanced Cell Diagnostics, Hayward, CA, USA) for 15 min at 40°C followed by 5% casein blocking buffer (20 min RT) and incubated with an antibody cocktail against CK7 and CK19 (mouse IgG2a anti-CK19 clone A53-BA2.26, Sigma Aldrich). Matched negative controls were conducted by omitting the target probe and primary antibody. Slides were Hoechst counterstained and mounted in Prolong Gold mounting media before whole-slide scanning in the VS200 Olympus slide scanner (Tokyo, Japan). Scanning was performed at 40x magnification with 30 ms exposure in the DAPI channel (455 nm), 80 ms exposure in the Cy3 channel (565 nm), 300 ms exposure in the Cy5 channel (670 nm), and 70 ms in Texas Red (615 nm). Image visualization settings (brightness and contrast) were differentially adjusted for image quantification and representative image acquisition. Artificial intelligence-assisted histology was conducted with the IF+FISH v2.1.5 module of HALO (Indica Labs). Cell segmentation and classification were followed by 10-μm band proximity analyses of osteocytes within 500μm of CK7/19^+^ cancer cells.

### Statistics*.*

Data were analyzed using GraphPad (GraphPad Software Inc, San Diego, CA, US). Differences in means were analyzed using a combination of unpaired *t*-test and ANOVA, followed by pairwise multiple comparisons (Tukey). Values were reported as means ± SD. P values ≤ 0.05 were considered statistically significant. Data analysis was performed in a blinded fashion.

### Study Approvals.

All animal procedures were performed following guidelines issued by the Institutional Animal Care and Use Committee at the University of Arkansas for Medical Sciences (AUP protocol #2022200000489). Institutional and national guides for the care and use of laboratory animals were followed for these studies. Collection and de-identification of human bone samples were coordinated by the UAMS Winthrop P. Rockefeller Cancer Institute Tissue Biorepository and Procurement Service (TBAPS) and approved by the UAMS Institutional Review Board (IRB protocol # 262940). Archived diagnostic transiliac biopsies collected at the Pathological Biobank at Odense University Hospital, Denmark, from four consented female patients with breast cancer metastatic bone disease were included in this analysis under approval from the National Committee on Health Research Ethics (S-20180057). All participants provided written, informed consent before study procedures occurred, with continuous consent ensured throughout participation.

## Data Availability

The sc-RNA-seq data generated in this manuscript were deposited in the NCBI SRA database under Bioproject PRJNA1033671. Other non-public datasets used and analyzed during the current study are available from the corresponding author upon reasonable request.

## Statement of significance

Breast cancer bone metastases cause bone destruction and are incurable. Herein, we unraveled that breast cancer cells reprogram osteocytes, resulting in premature senescence, and show that targeting cellular senescence alleviates bone destruction.

## References

[1] D’Oronzo S, Metastatic bone disease: Pathogenesis and therapeutic options: Up-date on bone metastasis management. J Bone Oncol. 2019;15:004–4. 30937279 10.1016/j.jbo.2018.10.004 PMC6429006

[2] Croucher PI, Bone metastasis: the importance of the neighbourhood. Nat Rev Cancer. 2016;16(6):373–86. 27220481 10.1038/nrc.2016.44

[3] Sung H, Global Cancer Statistics 2020: GLOBOCAN Estimates of Incidence and Mortality Worldwide for 36 Cancers in 185 Countries. CA Cancer J Clin. 2021;71(3):209–49. 33538338 10.3322/caac.21660

[4] Guise TA. The vicious cycle of bone metastases. J Musculoskelet Neuronal Interact. 2002;2(6):570–2. 15758398

[5] Paget S. The distribution of secondary growths in cancer of the breast. 1889. Cancer Metastasis Rev. 1989;8(2):98–101. 2673568

[6] Buenrostro D, The Bone Microenvironment: a Fertile Soil for Tumor Growth. Curr Osteoporos Rep. 2016;14(4):151–8. 27255469 10.1007/s11914-016-0315-2 PMC4927340

[7] Bonewald LF. The Amazing Osteocyte. J Bone Miner Res. 2011;26(2):229–38. 21254230 10.1002/jbmr.320 PMC3179345

[8] Sabol HM, Notch3 signaling between myeloma cells and osteocytes in the tumor niche promotes tumor growth and bone destruction. Neoplasia. 2022;28:100785. 35390742 10.1016/j.neo.2022.100785 PMC8990177

[9] Delgado-Calle J, Genetic deletion of Sost or pharmacological inhibition of sclerostin prevent multiple myeloma-induced bone disease without affecting tumor growth. Leukemia. 2017;31(12):2686–94. 28529307 10.1038/leu.2017.152 PMC5699973

[10] Delgado-Calle J, Bidirectional Notch Signaling and Osteocyte-Derived Factors in the Bone Marrow Microenvironment Promote Tumor Cell Proliferation and Bone Destruction in Multiple Myeloma. Cancer Res. 2016;76(5):1089–100. 26833121 10.1158/0008-5472.CAN-15-1703 PMC4775415

[11] Giuliani N, Increased osteocyte death in multiple myeloma patients: role in myeloma-induced osteoclast formation. Leukemia. 2012;26(6):1391–401. 22289923 10.1038/leu.2011.381

[12] Wang W, Prostate cancer promotes a vicious cycle of bone metastasis progression through inducing osteocytes to secrete GDF15 that stimulates prostate cancer growth and invasion. Oncogene. 2019;38(23):4540–59. 30755731 10.1038/s41388-019-0736-3 PMC9097780

[13] Sottnik JL, Tumor-induced pressure in the bone microenvironment causes osteocytes to promote the growth of prostate cancer bone metastases. Cancer Res. 2015;75(11):2151–8. 25855383 10.1158/0008-5472.CAN-14-2493 PMC4452392

[14] Sabol HM, Targeting Notch Inhibitors to the Myeloma Bone Marrow Niche Decreases Tumor Growth and Bone Destruction without Gut Toxicity. Cancer Res. 2021;81(19):5102–14. 34348968 10.1158/0008-5472.CAN-21-0524 PMC8488008

[15] McDonald MM, Inhibiting the osteocyte-specific protein sclerostin increases bone mass and fracture resistance in multiple myeloma. Blood. 2017;129(26):3452–64. 28515094 10.1182/blood-2017-03-773341 PMC5492093

[16] Hesse E, Sclerostin inhibition alleviates breast cancer-induced bone metastases and muscle weakness. JCI Insight. 2019;5(9). 10.1172/jci.insight.125543 PMC6538320 30965315

[17] Hemmatian H, Reorganization of the osteocyte lacuno-canalicular network characteristics in tumor sites of an immunocompetent murine model of osteotropic cancers. Bone. 2021;152:116074. 34174502 10.1016/j.bone.2021.116074

[18] Liu S, Osteocyte-Driven Downregulation of Snail Restrains Effects of Drd2 Inhibitors on Mammary Tumor Cells. Cancer Res. 2018;78(14):3865–76. 29769195 10.1158/0008-5472.CAN-18-0056 PMC6050076

[19] Cui YX, New Roles of Osteocytes in Proliferation, Migration and Invasion of Breast and Prostate Cancer Cells. Anticancer Res. 2016;36(3):1193–201. 26977015

[20] Tian Y, Osteocytic Connexin Hemichannels Modulate Oxidative Bone Microenvironment and Breast Cancer Growth. Cancers (Basel). 2021;13(24). 10.3390/cancers13246343 PMC8699531 34944962

[21] Roh HC, Simultaneous Transcriptional and Epigenomic Profiling from Specific Cell Types within Heterogeneous Tissues In Vivo. Cell Rep. 2017;18(4):1048–61. 28122230 10.1016/j.celrep.2016.12.087 PMC5291126

[22] Xiong J, Osteocytes, not Osteoblasts or Lining Cells, are the Main Source of the RANKL Required for Osteoclast Formation in Remodeling Bone. PLoS ONE. 2015;10(9):e0138189. 26393791 10.1371/journal.pone.0138189 PMC4578942

[23] Cords L, Cancer-associated fibroblast classification in single-cell and spatial proteomics data. Nat Commun. 2023;14(1):4294. 37463917 10.1038/s41467-023-39762-1 PMC10354071

[24] Han C, Biomarkers for cancer-associated fibroblasts. Biomark Res. 2020;8(1):64. 33292666 10.1186/s40364-020-00245-w PMC7661188

[25] Saul D, A new gene set identifies senescent cells and predicts senescence-associated pathways across tissues. Nat Commun. 2022;13(1):4827. 35974106 10.1038/s41467-022-32552-1 PMC9381717

[26] Fridman AL, and Tainsky MA. Critical pathways in cellular senescence and immortalization revealed by gene expression profiling. Oncogene. 2008;27(46):5975–87. 18711403 10.1038/onc.2008.213 PMC3843241

[27] Spatz JM, The Wnt-inhibitor Sclerostin is Up-regulated by Mechanical Unloading in Osteocytes in-vitro. J Biol Chem. 2015;290(27):16744–58. 25953900 10.1074/jbc.M114.628313 PMC4505423

[28] Bellido T, and Delgado-Calle J. Ex Vivo Organ Cultures as Models to Study Bone Biology. JBMR Plus. 2020;4(3). 10.1002/jbm4.10345 PMC7059827 32161838

[29] Chandra A, Targeted Reduction of Senescent Cell Burden Alleviates Focal Radiotherapy-Related Bone Loss. J Bone Miner Res. 2020;35(6):1119–31. 32023351 10.1002/jbmr.3978 PMC7357625

[30] Farr JN, Targeting cellular senescence prevents age-related bone loss in mice. Nat Med. 2017;23(9):1072–9. 28825716 10.1038/nm.4385 PMC5657592

[31] Agoro R, Single cell cortical bone transcriptomics define novel osteolineage gene sets altered in chronic kidney disease. Front Endocrinol (Lausanne). 2023;14:1063083. 36777346 10.3389/fendo.2023.1063083 PMC9910177

[32] Wang JS, Control of osteocyte dendrite formation by Sp7 and its target gene osteocrin. Nat Commun. 2021;12(1):6271. 34725346 10.1038/s41467-021-26571-7 PMC8560803

[33] Nookaew I, A framework for defining mesenchymal cell types associated with murine periosteal and endosteal bone. bioRxiv. 2023:2023.11.17.567528. 10.1016/j.jbc.2024.107158 PMC11007436 38479598

[34] Paic F, Identification of differentially expressed genes between osteoblasts and osteocytes. Bone. 2009;45(4):682–92. 19539797 10.1016/j.bone.2009.06.010 PMC2731004

[35] Mendoza-Villanueva D, Metastatic breast cancer cells inhibit osteoblast differentiation through the Runx2/CBFbeta-dependent expression of the Wnt antagonist, sclerostin. Breast Cancer Res. 2011;13(5):R106. 22032690 10.1186/bcr3048 PMC3262219

[36] Mastro AM, Breast cancer cells induce osteoblast apoptosis: a possible contributor to bone degradation. J Cell Biochem. 2004;91(2):265–76. 14743387 10.1002/jcb.10746

[37] Mercer RR, Metastatic breast cancer cells suppress osteoblast adhesion and differentiation. Clin Exp Metastasis. 2004;21(5):427–35. 15672867 10.1007/s10585-004-1867-6

[38] Bu G, Breast cancer-derived Dickkopf1 inhibits osteoblast differentiation and osteoprotegerin expression: implication for breast cancer osteolytic bone metastases. Int J Cancer. 2008;123(5):1034–42. 18546262 10.1002/ijc.23625 PMC3732167

[39] Eckhardt BA, Accelerated osteocyte senescence and skeletal fragility in mice with type 2 diabetes. JCI Insight. 2020;5(9). 10.1172/jci.insight.135236 PMC7253018 32267250

[40] Farr JN, Local senolysis in aged mice only partially replicates the benefits of systemic senolysis. J Clin Invest. 2023;133(8). 10.1172/JCI162519 PMC10104901 36809340

[41] Kim HN, Osteocyte RANKL is required for cortical bone loss with age and is induced by senescence. JCI Insight. 2020;5(19). 10.1172/jci.insight.138815 PMC7566701 32870816

[42] Martin TJ, and Johnson RW. Multiple actions of parathyroid hormone-related protein in breast cancer bone metastasis. Br J Pharmacol. 2021;178(9):1923–35. 31087800 10.1111/bph.14709 PMC8445224

[43] Prasanna PG, Therapy-Induced Senescence: Opportunities to Improve Anti-Cancer Therapy. J Natl Cancer Inst. 2021. 10.1093/jnci/djab064 PMC8486333 33792717

[44] Demaria M, Cellular Senescence Promotes Adverse Effects of Chemotherapy and Cancer Relapse. Cancer Discov. 2017;7(2):165–76. 27979832 10.1158/2159-8290.CD-16-0241 PMC5296251

[45] Carpenter VJ, Senolytics for Cancer Therapy: Is All That Glitters Really Gold? Cancers (Basel). 2021;13(4). 10.3390/cancers13040723 PMC7916462 33578753

[46] Wang L, Exploiting senescence for the treatment of cancer. Nat Rev Cancer. 2022;22(6):340–55. 35241831 10.1038/s41568-022-00450-9

[47] Maugeri A, Targets Involved in the Anti-Cancer Activity of Quercetin in Breast, Colorectal and Liver Neoplasms. Int J Mol Sci. 2023;24(3). 10.3390/ijms24032952 PMC9918234 36769274

[48] Bahman F, Enhanced Anticancer Activity of Nanoformulation of Dasatinib against Triple-Negative Breast Cancer. J Pers Med. 2021;11(6). 10.3390/jpm11060559 PMC8234460 34204015

[49] Wang L, High-Throughput Functional Genetic and Compound Screens Identify Targets for Senescence Induction in Cancer. Cell Rep. 2017;21(3):773–83. 29045843 10.1016/j.celrep.2017.09.085

[50] Luo X, Stromal-Initiated Changes in the Bone Promote Metastatic Niche Development. Cell Rep. 2016;14(1):82–92. 26725121 10.1016/j.celrep.2015.12.016 PMC4706805

[51] Liu D, and Hornsby PJ. Senescent human fibroblasts increase the early growth of xenograft tumors via matrix metalloproteinase secretion. Cancer Res. 2007;67(7):3117–26. 17409418 10.1158/0008-5472.CAN-06-3452

[52] Saleh T, Clearance of therapy-induced senescent tumor cells by the senolytic ABT-263 via interference with BCL-X(L)-BAX interaction. Mol Oncol. 2020;14(10):2504–19. 32652830 10.1002/1878-0261.12761 PMC7530780

[53] Collado M, and Serrano M. Senescence in tumours: evidence from mice and humans. Nat Rev Cancer. 2010;10(1):51–7. 20029423 10.1038/nrc2772 PMC3672965

[54] Plotkin LI, Prevention of osteocyte and osteoblast apoptosis by bisphosphonates and calcitonin. J Clin Invest. 1999;104(10):1363–74. 10562298 10.1172/JCI6800 PMC409837

[55] Bivi N, Deletion of Cx43 from osteocytes results in defective bone material properties but does not decrease extrinsic strength in cortical bone. Calcif Tissue Int. 2012;91(3):215–24. 22865265 10.1007/s00223-012-9628-z PMC3729333

[56] Farr JN, Identification of Senescent Cells in the Bone Microenvironment. J Bone Miner Res. 2016. 10.1002/jbmr.2892 PMC5289710 27341653

[57] Stuart T, Comprehensive Integration of Single-Cell Data. Cell. 2019;177(7):1888–902.e21. 31178118 10.1016/j.cell.2019.05.031 PMC6687398

[58] Hao Y, Integrated analysis of multimodal single-cell data. Cell. 2021;184(13):3573–87.e29. 34062119 10.1016/j.cell.2021.04.048 PMC8238499

[59] Hafemeister C, and Satija R. Normalization and variance stabilization of single-cell RNA-seq data using regularized negative binomial regression. Genome Biol. 2019;20(1):296. 31870423 10.1186/s13059-019-1874-1 PMC6927181

[60] Lancichinetti A, and Fortunato S. Community detection algorithms: A comparative analysis. Physical Review E. 2009;80(5):056117. 10.1103/PhysRevE.80.056117 20365053

[61] Finak G, MAST: a flexible statistical framework for assessing transcriptional changes and characterizing heterogeneity in single-cell RNA sequencing data. Genome Biol. 2015;16:278. 26653891 10.1186/s13059-015-0844-5 PMC4676162

[62] Väremo L, Enriching the gene set analysis of genome-wide data by incorporating directionality of gene expression and combining statistical hypotheses and methods. Nucleic Acids Res. 2013;41(8):4378–91. 23444143 10.1093/nar/gkt111 PMC3632109

[63] Youlten SE, Osteocyte Transcriptome Mapping Identifies a Molecular Landscape Controlling Skeletal Homeostasis and Susceptibility to Skeletal Disease. bioRxiv. 2020:2020.04.20.051409. 10.1038/s41467-021-22517-1 PMC8100170 33953184

[64] Fu Q, Reduced OPG expression by osteocytes may contribute to rebound resorption after denosumab discontinuation. JCI Insight. 2023. 10.1172/jci.insight.167790 PMC10561722 37581932

[65] Swanson EC, Higher-order unfolding of satellite heterochromatin is a consistent and early event in cell senescence. J Cell Biol. 2013;203(6):929–42. 24344186 10.1083/jcb.201306073 PMC3871423

[66] Saul D, Modulation of fracture healing by the transient accumulation of senescent cells. Elife. 2021;10. 10.7554/eLife.69958 PMC8526061 34617510

[67] Schindelin J, Fiji: an open-source platform for biological-image analysis. Nat Methods. 2012;9(7):676–82. 22743772 10.1038/nmeth.2019 PMC3855844

